# Knowledge, attitudes and practices towards glycemic control among persons with diabetes mellitus at two tertiary hospitals in Uganda

**DOI:** 10.4314/ahs.v24i1.22

**Published:** 2024-03

**Authors:** Daphine Ninsiima, Timothy Lwanga, Gerald Kevin Oluka, Emmanuel Oguti Okodoi, William Aine, Henry Lwibasira, Brian Ndibarema, Hama Abaho, Ronald Olum, Irene Andia-Biraro, Felix Bongomin

**Affiliations:** 1 School of Medicine, College of Health Sciences, Makerere University, Kampala, Uganda; 2 Department of Medicine, Nsambya Hospital, Kampala, Uganda; 3 Department of Medical Microbiology, Faculty of Medicine, Gulu University, Gulu, Uganda

**Keywords:** Diabetes mellitus, glycemic control, HbA_1_C, KAPs

## Abstract

**Background:**

Intensive glycaemic control reduces the risk of microvascular complications in persons with diabetes mellitus (DM). Owing to limited data available, we aimed to determine the knowledge, attitude, and practices (KAPs) toward glycaemic control among Ugandans with DM at two large tertiary healthcare facilities.

**Methods:**

A cross-sectional study was conducted among persons with DM attending outpatient clinics at Kiruddu National Referral Hospital (KNRH) and Mulago National Specialized Hospital (MNSH) between March and April 2022. Eligible participants provided written informed consent and were recruited through a systematic sampling technique and relevant data was collected using a pretested, interviewer-administered, semi-structured questionnaire.

**Results:**

Of the 452 participants, 318 (70.4%) were females. The median age was 52 years (IQR: 45–60 years), with more than two-thirds diagnosed with DM at ≥36 years (69.8%, n=310). Overall, 274 participants (60.6%) had good knowledge on glycemic control. At multivariable logistic regression analysis, good knowledge about glycaemic control was significantly associated with having received training on glycaemic control (aOR: 2.3, 95% CI: 1.4 – 3.7, p=0.002), level of education: diploma (aOR: 4.3, 95% CI: 1.1 – 17.8, p=0.042), degree aOR: 4.9, 95% CI: 1.0 – 23.1, p=0.046) compared to informal education, and nearest distance from the health facility (aOR: 3.1, 95% CI: 1.0 – 9.6, p=0.047)

**Conclusions:**

More than half of the patients had good knowledge about glycaemic control and this was associated with level of education, distance from the health facility and having received training. Further studies assessing the correlations between actual level of glycaemic control and patient related KAPs are recommended.

## Introduction

Diabetes mellitus (DM) is a heterogeneous group of metabolic disorders characterized by chronic hyperglycemia resulting from defects in insulin secretion, insulin action, or both 1. The metabolic derangements in DM consequently lead to micro-and macro-vascular complications such as diabetic neuropathy and cardiovascular accidents^2^. DM is one of the top ten leading causes of death in the world today, with an estimated 1.5 million people dying each year, due to its related complications 3. Globally, the number of people living with DM has continued to increase steadily; estimated to have increased from 108 million in 1980, to 422 million in 2014. At the end of 2021, about 537 million adults aged 20-79 years were living with DM, and the number is projected to increase to 643 million by 2030, and 784 million by 2045 3,4. This clearly represents a dire trend of events that warrants close attention and decisive intervention. 4 in 5 of adults living with DM are in low- and middle-income countries, Uganda inclusive; with a 1.6% prevalence of DM, representing close to 500,000 people of the total population 5. A recent systematic review and meta-analysis has shown a significant burden of diabetes complications in low-resource settings. 6

Good glycemic control (GC) is characterized by a glycosylated hemoglobin A1c(HbA1c) concentration of < 7% (53 mmol/mol) and this is the ultimate goal of DM management 7. Good GC prevents or slows down the progression of the long-term complications of DM. It is highly recommended for persons with DM to have at least two HbA1c tests per year in order to have an early prediction of microvascular complications of DM 8. Improving glycemic control requires that patients actively participate in decisions about how to best live with the disease and adapt to the realities of self-care. Poor knowledge about glycemic control makes it difficult for patients to participate in shared decision-making (SDM) 9. In Uganda, a study of patients with DM at Mbarara Regional Referral found a high prevalence of poor GC with a high majority (84.3%) having an HbA1C ≥7%. The poor GC was more prevalent among participants aged 25–60 years, or above compared to the youth, 18–24 years of age. Age was therefore a significant factor associated with poor GC 10.

An earlier study found out that most patients with hyperglycaemia in Uganda are unaware of their glycemic status, increasing the likelihood of presenting late with complications 5. According to Patrick et al. (2021), 81.7% of the patients with poor GC, did not adhere to diet recommendations, and 90% of them did not adhere to physical exercise recommendations. This shows a high prevalence of poor practices in glycemic control among diabetic patients in Uganda. This finding is also consistent with the fact that most (over 80%) of the diabetic patients in Uganda have had at least one diabetic complication 11. Much as Patrick et al. (2021) attributed the poor glycemic control to lack of knowledge among the study population, most of whom were illiterate and had a very low level of education, it can sometimes be difficult be to make such a straight-forward connection since other researchers have often found a similar lack of knowledge regarding glycemic control even among university students 12.

The current situation points towards glaring inadequacies in patients' knowledge, attitudes, and practices (KAPs) towards glycemic control, and it was thus imperative to study the matter and find out the nature and extent of the inadequacies and determine what factors might be contributing to this rapidly emerging public-health concern, and thereby enact necessary policies and protocols for better patient therapeutic outcomes. Therefore, this study aimed to assess the KAPs towards GC, and associated factors among patients with DM at Kiruddu National Referral Hospital (KNRH) and Mulago National Specialized Hospital (MNSH).

## Methods

### Study design

We conducted a healthcare facility-based cross-sectional survey amongst patients with diabetes mellitus between March and April 2022. The study was quantitative in approach, using a pretested interviewer-based questionnaire.

### Study area and setting

The study was conducted at two diabetes clinics of KNRH and MNSH, Uganda's top-most public tertiary hospitals, located in Kampala city. Bat the time of the survey, both clinics had an average attendance of about 100 to 150 patients per clinic day. KNRH diabetes clinic runs every Wednesday and MNSH clinic runs every Friday. The patients are mainly those with diabetes and/or other endocrine disorders from the central region of Uganda and occasionally referrals from across the country. Both clinics have resident endocrinologists/diabetologists, with additional work force being derived from a pool of general internal medicine physicians, senior house officers (SHOs), and medical officers in addition to general and specialized nurses.

### Study population

Participants in this study were patients aged 18 years and above, diagnosed with diabetes mellitus, and attending the diabetes clinics at either KNRH or MNSH during the period of the study.

### Sample size and sampling techniques

A sample size of 422 patients was calculated using the survey formula by Kish-Leslie (1965); with an awareness proportion (P) of 50%, precision error (d) of 5% at 95% confidence interval (Z), and a non-response rate of 10%. The sample size was equally distributed to the two clinics such that 211 patients were interviewed from each of the clinics. The study population was obtained by systematic sampling, selecting every 3rd participant on the sampling frame meeting the inclusion criteria.

### Study variables

The independent variables included were patient demographics such as age, gender, education, occupation, co-morbidities. The dependent variables included: knowledge, attitudes, and practices towards glycemic control among the diabetic patients.

### Participant selection criteria

The inclusion criteria considered patients who were aged 18 years and above, diagnosed with diabetes mellitus, and were attending the diabetes clinics at KNRH and MNSH for the duration of the study. Informed consent was sought before participation in the study. Excluded participants were patients with mental health illness who were unable to comprehend the contents of the questionnaire.

### Data collection

Study participants were subjected to a questionnaire-based interview. The questionnaire was designed and deployed online using KoBo Toolbox. Data assistants collected the data by interviewing patients and filling in the responses into the online questionnaires. The KAPs questionnaire comprised 12 questions on demographic characteristics of the participants, 12 questions assessing the knowledge of participants regarding glycemic control, 12 questions assessing the attitude towards glycemic control, and 10 questions assessing the participants' self care practices in achieving glycemic control. The tool used to measure KAPs was adopted from previous studies^13^ ^14^ ^15^ with considerable improvements and modifications.

### Quality control

The questionnaire used in data collection was pretested on 5% of the patients (21 patients) and the identified necessary corrections were made before administering the tool to the final study participants. The patients who participated in this pretesting did not thereafter, participate in the study. The link only shared amongst the team collecting data and only the PI had access to the collected data. The questionnaire had check points that ensure that only completed forms could be submitted. Each patient was interviewed only once to exclude duplication of data.

### Data management and analysis

After completing the data collection process, the entries were downloaded. The collected data was cleaned and coded using Microsoft Excel 2016. The coded data was exported to STATA software version 17 for analysis according to the objectives of the study. A bloom's cut-off of ≥ 80% was used to define sufficient knowledge. Logistic regression analysis was performed to identify factors associated with poor glycemic control. P< 0.05 was considered statistically significant at a confidence interval of 95%.

### Ethical considerations

Approval to carry out this study was sought from the Mulago Hospital Research and Ethics Committee (MHREC 2169). Mulago Hospital is a teaching for the Makerere University College of Health Sciences and was one of the 2 study sites. The study and its purpose were explained to each study-participant and signed informed-consent obtained from each. All study-participant's information was kept confidential and anonymous using codes. Administrative clearance was sought from both study sites.

## Results

### Characteristics of the study participants

A total of 452 patients diagnosed with DM attending the DM clinic at Kiruddu National Referral Hospital were interviewed. The median age of the participants was 52 years (interquartile range: 45 – 60 years) and the majority were aged 36 – 59 years (63.5%, n=287), female (70.4%, n=318) and living in urban residences (73.3%, n=329). Almost half of the participants (47%, n=189) were earning less than 100,000 UGX (∼28 USD) per month whereas up to 82.8% (n=370) were living within 5 kilometers from the nearest health facility. Table 1 summarizes the social and demographic characteristics of the participants.

**Table 1:** Characteristics of study participants

Characteristics	Frequency	Percentage
**Age: Median years (interquartile range)**	52	45 - 60
**Age category**		
18 – 35 years	52	11.5
36 – 59 years	287	63.5
≥ 60 years	113	25.0
**Gender**		
Female	318	70.4
Male	134	29.7
**Nationality (n=451)**		
Uganda	440	97.4
Non-Ugandan	11	2.6
**Marital status**		
Single	40	8.9
Married	279	61.7
Divorced	66	14.6
Widowed	67	14.8
**Education level**		
Informal	58	12.8
Primary	177	39.2
O-level	149	33.0
A' level/equivalent	26	5.8
Diploma	24	5.3
Degree	18	4.0
**Employment status**		
Employed	261	57.7
Unemployed	191	42.3
**If employed, employment type (n=261)**		
Self-employed	191	73.2
Private sector	46	17.6
Government sector	16	6.1
Retired	6	2.3
Not specified	2	0.8
**Estimated monthly income (n=402)**		
Less than 100,000	189	47.0
Between 100,001-300,000	102	25.4
Between 300,001-500,000	65	16.2
Greater than 500,000	46	11.4
**Religion (n=450)**		
Catholic	122	27.1
Muslims	125	27.8
Anglican	121	26.9
Pentecostal	73	16.2
Seventh Day Adventist	5	1.1
Other(specify) ^a^	4	0.9
**District of residence (n=451)**		
Kampala	209	46.3
Wakiso	189	41.9
Mukono	11	2.4
Others	42	9.3
**Residence (n=449)**		
Urban	329	73.3
Rural	120	26.7
**Nearest health facility (n=450)**		
HC II	11	2.4
HCIII	46	10.2
HCIV	73	16.2
Clinic	94	20.9
District hospital	42	9.3
Regional referral hospital	184	40.9
**Distance from the facility in km (n=447)**		
0 to 5 km	370	82.8
6 to 10 km	48	10.7
Greater than 10km	29	6.5

Note: *a* – Jehovah Witness (1), Orthodox (1), Atheist (1) and *Mungumwena* (1).

**Figure 1:**
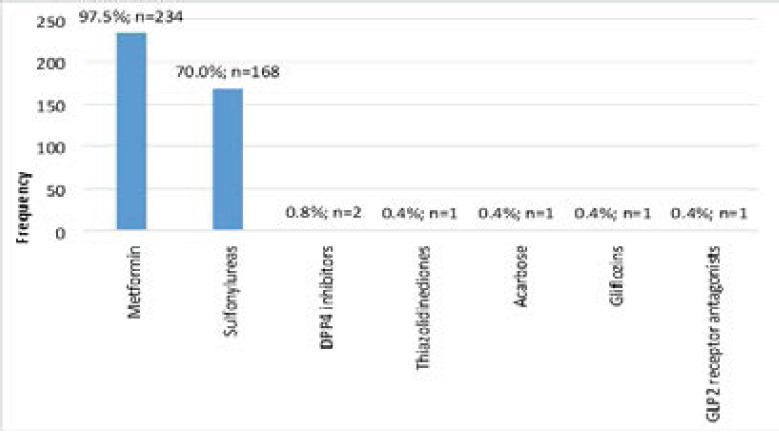
Oral antihyperglycemic use among the study participants (n=240)

**Figure 2:**
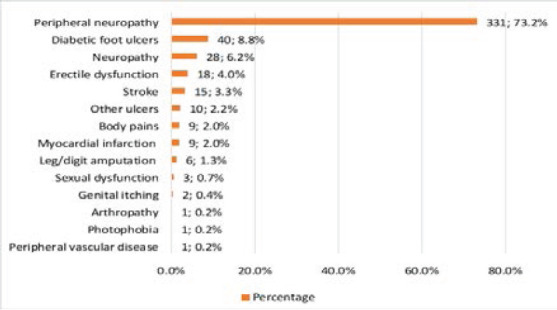
Complications reported in the study participants

Table 2 describes DM-related characteristics among the participants. The median age of diagnosis of DM was 43 years (IQR: 35 – 51 years), and more than two-thirds were diagnosed at ≥36 years (69.8%, n=310). Regarding the current treatment, 53.2% (n=240) were on oral anti-hyperglycemics only whereas 15.7% (n=71) and 31.0% (n=140) were on insulin only or both, respectively. Among patients on insulin only, premixed insulin was the most common regimen (n=65, 91.6%) whereas metformin (97.5%, n=234) and sulfonylureas (70.0%, n=168) were the most frequently used drugs among those on oral anti-hyperglycemics only (Figure 1). Almost two-thirds of the patients had comorbid hypertension (63.1%, n=285) and only 6.9% (n=31) had HIV. Up to 73.2% (n=331) of the patients had peripheral neuropathy and about 6 participants (1.3%) had leg/digit amputation secondary to DM complications (Figure 2).

**Table 2:** Diabetes mellitus related characteristics among the study participants

Characteristics	Frequency	Percentage
**Age at diagnosis of DM: Median (IQR) years**	43	35 - 51
**Age category at diagnosis of DM**		
<18 years	15	3.4
18-36 years	119	26.8
>36 years	310	69.8
**Duration with diabetes (n=448)**		
<5 years	167	37.3
5 to 10 years	143	31.9
>10 years	138	30.8
**Family history of DM**		
No	181	40.0
Yes	271	60.0
**Current treatment (n=451)**		
Both insulin and oral antihyperglycemics	140	31.0
Insulin only	71	15.7
Oral antihyperglycemics only	240	53.2
**Specify insulin regimen (n=71)**		
Basal-bolus insulin	6	8.5
Twice daily (premixed insulin)	65	91.6
**Received training on glycemic control (n=451)**		
No	111	24.6
Yes	340	75.4
**Owning a glucometer**		
No	273	60.4
Yes	179	39.6
**Knowledge of glucometer usage (n=178)**		
No	30	16.9
Yes	148	83.2
**Blood sugar monitoring (n=449)**		
Never	6	1.3
At least once a day	44	9.8
At least once a month	89	19.8
At least once a week	102	22.7
Only reviews in DM clinic	192	42.8
When not feeling well	16	3.6
**Comorbidities**		
Hypertension	285	63.1
HIV	31	6.9
Gastritis/Peptic ulcerative disease	27	6.0
Asthma	6	1.3
Tumor	2	0.4
Allergies	2	0.4
Others (specify)	44	9.7
None	116	25.7
**How often do you go for clinic visits in a month**		
Every 1 month	150	33.2
Every 2 months	70	15.5
Every 3 months	173	38.3
Every 4 months	1	0.2
Every six months	1	0.2
On appointment	12	2.7
Not specified	35	7.7
Not been attending clinic	10	2.2

### Knowledge on diabetes mellitus

Table 3 summarizes the responses of the participants towards questions assessing knowledge on diabetes mellitus. The median knowledge score was 75% (interquartile range: 58% - 83%). Participants who scored above average (75% or higher) were considered to have good knowledge. Overall, 274 participants (60.6%) had good knowledge on diabetes mellitus.

**Table 3:** Knowledge of the participants on diabetes mellitus

Knowledge on diabetes mellitus (n=452)	Yes: n (%)	No: n (%)	Unsure: n (%)
Diabetes is caused by too much sugar	101 (22.3)	214 (47.3)	137 (30.3)
Diabetes is a single entity	56 (12.4)	232 (51.3)	164 (36.3)
All patients with DM require insulin injection	31 (6.9)	365 (80.8)	56 (12.4)
Patients with diabetes can have low blood sugar levels.	376 (83.2)	40 (8.8)	36 (8)
DM is hereditary	261 (57.7)	103 (22.8)	88 (19.5)
High blood pressure can be a complication of DM	323 (71.5)	46 (10.2)	83 (18.4)
People with diabetes require regular monitoring of blood sugars	423 (93.6)	16 (3.5)	13 (2.9)
Diabetes affects only one organ	54 (11.9)	340 (75.2)	58 (12.8)
Diabetes has no long-term complications	145 (32.1)	275 (60.8)	32 (7.1)
Regular exercise is bad for people with DM	50 (11.1)	373 (82.5)	29 (6.4)
Very high blood sugar may require hospitalization	367 (81.2)	60 (13.3)	25 (5.5)
Smoking is bad for patients with DM	368 (81.4)	12 (2.7)	72 (15.9)

### Factors a ssociated with knowledge on diabetes mellitus

Good knowledge was significantly associated with current age (p=0.001), marital status (p=0.009), level of education (=0.042), employment status (p=0.007), religion (p=0.030), age at diagnosis (p<0.001), duration with DM diagnosis (p=0.019), and receiving training on glycemic control (p<0.001), Table 4.

**Table 4:** Distribution of knowledge on diabetes among the study participants

Variable	Poor	Good	P-value
**Gender**			
Female	124 (39)	194 (61)	0.795
Male	54 (40.3)	80 (59.7)	
**Age in years**			
18 - 35	14 (26.9)	38 (73.1)	0.001
35 - 59	104 (36.2)	183 (63.8)	
60+	60 (53.1)	53 (46.9)	
**Nationality**			
Other	6 (50)	6 (50)	0.445
Ugandan	172 (39.1)	268 (60.9)	
**Marital status**			
Divorced	23 (34.8)	43 (65.2)	0.009
Married	106 (38)	173 (62)	
Single	11 (27.5)	29 (72.5)	
Widowed	38 (56.7)	29 (43.3)	
**Education level**			
Informal	29 (50.0)	29 (50.0)	0.042
Primary	76 (42.9)	101 (52.1)	
Secondary	64 (36.6)	111 (63.4)	
Diploma	5 (20.8)	19 (79.2)	
Degree	4 (22.2)	14 (77.8)	
**Employment status**			
Employed	89 (34.1)	172 (65.9)	0.007
Unemployed	89 (46.6)	102 (53.4)	
**Estimated monthly income**			
Between 100,001-300,000	39 (38.2)	63 (61.8)	0.742
Between 300001-500000	22 (33.8)	43 (66.2)	
Greater than 500000	15 (32.6)	31 (67.4)	
Less 100,000	75 (39.7)	114 (60.3)	
**Religion**			
Anglican	47 (38.8)	74 (61.2)	0.030
Catholic	45 (36.9)	77 (63.1)	
Muslims	47 (37.6)	78 (62.4)	
Other	17 (70.8)	7 (29.2)	
Pentecostal	21 (36.2)	37 (63.8)	
**Residence**			
Rural	49 (40.8)	71 (59.2)	0.668
Urban	127 (38.6)	202 (61.4)	
**Nearest health facility**			
Clinic	43 (45.7)	51 (54.3)	0.643
District hospital	13 (31)	29 (69)	
HC II	4 (36.4)	7 (63.6)	
HCIII	16 (34.8)	30 (65.2)	
HCIV	30 (41.1)	43 (58.9)	
Regional referral	71 (38.6)	113 (61.4)	
**Distance from health facility (km)**			
0 to 5	148 (40)	222 (60)	0.111
6 to 10	20 (41.7)	28 (58.3)	
Greater than 10	6 (20.7)	23 (79.3)	
**Age at diagnosis**			
<18	4 (26.7)	11 (73.3)	0.000
18 - 35	27 (25.2)	80 (74.8)	
36 - 59	117 (40.6)	171 (59.4)	
60+	26 (76.5)	8 (23.5)	
**Duration with DM diagnosis (years)**			
0 - 5	78 (46.7)	89 (53.3)	0.019
6 to 10	53 (37.1)	90 (62.9)	
Greater than 10	43 (31.2)	95 (68.8)	
**Family history of DM**			
No	78 (43.1)	103 (56.9)	0.187
Yes	100 (36.9)	171 (63.1)	
**Received training on glycemic control**			
No	60 (54.1)	51 (45.9)	0.000
Yes	118 (34.7)	222 (65.3)	

Participants ≥60 years (COR: 0.3, 95% CI: 0.2 – 0.7), widows/widowers (COR: 0.3, 95% CI: 0.1 – 0.7), those diagnosed with DM ≥60 years (COR: 0.1, 95% CI: 0.0 – 0.5, p=0.002) and participants from other religions (COR: 0.3, 95% CI: 0.1 – 0.7, p=0.006) were less likely to have good knowledge on diabetes mellitus at binary logistic regression analysis (Table 5). Conversely, participants with diploma (COR: 3.8, 95% CI: 1.3 – 11.5, p=0.045), degree education (COR: 3.5, 95% CI: 1.0 – 11.9, p=0.045), employment (COR: 1.7, 95% CI: 1.2 – 2.5, p=0.007), those living with DM for more than 10 years (COR: 1.9, 95% CI: 1.2 – 3.1, p=0.006), and patients who received training on glycemic control (COR: 2.2, 95% CI: 1.4 – 3.4, p<0.001) were more likely to have good knowledge on diabetes.

**Table 5:** Factors associated with knowledge on diabetes mellitus among the study participants

Variable	Crude odds ratio (95% CI)	P-value	Adjusted odds ratio (95% CI)	P-value
**Gender**				
Female	1.0			
Male	0.9 (0.6 - 1.4)	0.795		
**Age in years**				
18 - 35	1.0		1.0	
35 - 59	0.6 (0.3 - 1.3)	0.197	0.5 (0.2 - 1.4)	0.179
60+	0.3 (0.2 - 0.7)	0.002	0.3 (0.1 - 1.1)	0.080
**Nationality**				
Other	1.0			
Ugandan	1.6 (0.5 - 4.9)	0.449		
**Marital status**				
Married	1.0		1.0	
Divorced	0.7 (0.3 - 1.7)	0.433	1.4 (0.7 - 2.6)	0.335
Single	0.6 (0.3 - 1.3)	0.201	0.9 (0.4 - 2.3)	0.880
Widowed	0.3 (0.1 - 0.7)	0.004	0.7 (0.4 - 1.4)	0.314
**Education level**				
Informal	1.0		1.0	
Primary	1.3 (0.7 - 2.4)	0.349	1.1 (0.6 - 2.1)	0.789
Secondary	1.7 (1 - 3.2)	0.072	1.5 (0.7 - 2.9)	0.271
Diploma	3.8 (1.3 - 11.5)	0.019	4.3 (1.1 - 17.8)	0.042
Degree	3.5 (1 - 11.9)	0.045	4.9 (1 - 23.1)	0.046
**Employment status**				
Unemployed	1.0		1.0	
Employed	1.7 (1.2 - 2.5)	0.007	1.4 (0.9 - 2.2)	0.136
**Estimated monthly income**				
Less 100,000	1.0			
Between 100,001-300,000	1.1 (0.6 - 1.7)	0.809		
Between 300001-500000	1.3 (0.7 - 2.3)	0.404		
Greater than 500000	1.4 (0.7 - 2.7)	0.377		
**Religion**				
Anglican	1.0			
Catholic	1.1 (0.6 - 1.8)	0.753		
Muslims	1.1 (0.6 - 1.8)	0.841		
Other	0.3 (0.1 - 0.7)	0.006		
Pentecostal	1.1 (0.6 - 2.1)	0.734		
**Residence**				
Rural	1.0			
Urban	1.1 (0.7 - 1.7)	0.668		
**Nearest health facility**				
HCII	1.0			
HCIII	1.1 (0.3 - 4.2)	0.921		
HCIV	0.8 (0.2 - 3)	0.766		
Clinic	0.7 (0.2 - 2.5)	0.556		
District hospital	1.3 (0.3 - 5.1)	0.732		
Regional referral	0.9 (0.3 - 3.2)	0.883		
**Distance from health facility (km)**				
0 to 5	1.0		1.0	
6 to 10	0.9 (0.5 - 1.7)	0.825	1.0 (0.5 - 2.1)	0.920
Greater than 10	2.6 (1 - 6.4)	0.046	3.1 (1 - 9.6)	0.047
**Age at diagnosis**				
<18	1.0		1.0	
18 - 35	1.1 (0.3 - 3.7)	0.905	1.4 (0.4 - 5.5)	0.606
36 - 59	0.5 (0.2 - 1.7)	0.289	0.9 (0.2 - 3.9)	0.933
60+	0.1 (0 - 0.5)	0.002	0.3 (0 - 1.7)	0.173
**Duration with DM diagnosis (years)**				
0 - 5	1.0		1.0	
6 to 10	1.5 (0.9 - 2.3)	0.087	1.6 (1.0 - 2.8)	0.066
Greater than 10	1.9 (1.2 - 3.1)	0.006	1.8 (1.0-3.3)	0.070
**Family history of DM**				
No	1.0		1.0	
Yes	1.3 (0.9 - 1.9)	0.187	1.2 (0.8 - 1.8)	0.473
**Received training on glycemic control**				
No	1.0		1.0	
Yes	2.2 (1.4 - 3.4)	0.000	2.3 (1.4 - 3.7)	0.002

At multivariate logistic regression (Table 5), only education level, training on glycemic control and distance from health facility were significantly associated with good knowledge. Participants with diploma (AOR: 4.3, 95% CI: 1.1 – 17.8, p=0.042), degree (AOR: 4.9, 95% CI: 1.0 – 23.1, p=0.046), training in glycemic control (AOR: 2.3, 95% CI: 1.4 – 3.7, p=0.002) and those living more than 10 kilometers away from health facility (AOR: 3.1, 95% CI: 1.0 – 9.6, p=0.047) were significantly more likely to have good knowledge than their respective counterparts.

### Attitudes towards glycemic control

Majority of the participants believed that glycemic control is necessary for DM (97.3%) and prolongs life expectancy (92.9%). A greater number also believed that regular exercise (91.6%) and fruits and vegetables (90.9%) are good for glycemic control. More than two-thirds of the participants recognized smoking (77.9%) and alcohol consumption (80.1%) as factors associated with poor glycemic control and DM complications, respectively. On the other hand, up to 49.6% (n=224) believed that antidiabetic drugs (insulin or metformin) have harmful effects to the body. Table 6 describes the attitudes of the participants towards glycemic control.

**Table 6:** Attitudes of the participants towards glycemic control

Attitude	Yes: n (%)	No: n (%)	Unsure: n (%)
Glycemic control is necessary for DM	440 (97.3)	2 (0.4)	10 (2.2)
Regular exercise can help control sugar levels	414 (91.6)	6 (1.3)	32 (7.1)
Smoking causes poor glycemic control	352 (77.9)	7 (1.5)	93 (20.6)
Blood pressure control is necessary for glycemic control	352 (77.9)	22 (4.9)	78 (17.3)
Glycemic control prolongs life expectancy	420 (92.9)	11 (2.4)	21 (4.6)
Alternative treatments are good for glycemic control	150 (33.2)	175 (38.7)	127 (28.1)
Dietary alone glycemic control is better than medication with diet glycemic control	55 (12.2)	319 (70.6)	78 (17.3)
Fruits and vegetables are good for glycemic control	411 (90.9)	14 (3.1)	27 (6)
Alcohol can increase complications of diabetes	362 (80.1)	21 (4.6)	69 (15.3)
Insulin or metformin has harmful effects on the body	224 (49.6)	111 (24.6)	117 (25.9)
Traditional treatments are better than modern treatments in management of DM	30 (6.6)	320 (70.8)	102 (22.6)

### Practices towards glycemic control

Table 7 summarizes the practices of the patients towards glycemic control. The participants reported eating vegetables about 4 times a week (IQR: 3 – 7 times) and carrying out physical exercises 7 times a week (IQR: 1 – 7 times). Majority practiced medication adherence (83.2%) and body weight control (72.1%). About half (49.6%) reported having regular blood sugar checkups whereas 28.1% and 36.1% practiced eye care and foot care, respectively. Only 4.4% and 15.7% were currently smoking and drinking alcohol, respectively.

**Table 7:** Practices of the participants towards glycemic control

Practices	Frequency	Percentage
How often do you eat vegetables a week: median (IQR)	4	3 – 7
How often do you exercise per week: median (IQR)	7	1 – 7
Medication adherence	376	83.2
Maintenance /control of body weight	326	72.1
Regular blood sugar check-ups	224	49.6
Cigarette smoking	20	4.4
Extra sugar/salt on regular diet	79	17.5
Drinks alcohol	71	15.7
Eats food in time	260	57.5
Eye care	127	28.1
Foot care	163	36.1

## Discussion

This study aimed to assess the KAPs of patients with DM for the first time in Uganda from 2 NRHs. Results from this study revealed that 60.6% (274) of the participants had good knowledge of DM. More than 50% of the participants knew what DM is and about its the heterogeneity (51.3%), the different associated factors (81.4%) and complications of DM (60.8%), as well as various practices recommended for adequate GC (82.5%). Many attributed this to the different training sessions about DM usually offered to them on every clinic visit day by the physicians and nurses. These findings were higher as compared to studies done in Ethiopia where only 58.3% knew the cause of DM, 49.6% did not know about any complications of DM and only 59.8% knew about the associated factors of DM 13. However, comparably the knowledge was lower compared to similar studies done in Sri Lanka (77%)^16^. This was probably due to the low level of education among the Ugandan DM population. It has been argued that educating patients on their disease was an effective strategy to reduce complications of T2DM and achieve improved control over blood glucose 14.

When patients possess knowledge regarding their condition, the associated factors they face and what practices they need to undertake to achieve good GC, their attitudes and behaviors are more likely to be modified to produce positive health outcomes 17. Therefore, all the three factors (KAPs) are important in achieving good GC, since it has been found that even among DM populations with a good attitude towards GC, lack of the requisite knowledge was always a limiting factor in achieving good GC 18. Achieving an optimal level of glycemic control among patients requires adherence and cooperation from the patients, which in turn requires that at the bare minimum, patients possess the requisite knowledge, positive attitude and carry out proper practices necessary for glycemic control 13. Probably, higher knowledge could improve attitude and practice of the patients with regard to their disease^14^.

Regarding the attitude towards glycemic control, of all the participants, 440 (97.3%) of them believed that GC was very necessary for DM and importantly prolongs life expectancy (92.9%). A greater number also believed that regular exercise (91.6%) and fruits and vegetables (90.9%) are good for glycemic control. More than two-thirds of the participants recognized smoking (77.9%) and alcohol consumption (80.1%) as factors associated with poor glycemic control and DM complications, respectively. On the other hand, up to 49.6% (n=224) believed that antidiabetic drugs (insulin or metformin) have harmful effects to the body.

Among the 452 participants, 70.6% of them also believed that for adequate control of blood glucose, both medications and dietary considerations were important rather than diet alone. This finding was higher that similar studies done in Ethiopia (35.7%) 13 and Pakistan (68%)^19^. This could still be attributed the health education provided to the patients regarding medication adherence and proper nutrition. About 33.2% of the participants considered alternative treatments for GC and only 6.6% of the participants believed that traditional medicines had a superiority over the convection medicines in the management of DM. These derived their conclusion from pervious experiences with the traditional medicines. However, most of the participants had confidents in the convectional medicines as being more effective in managing DM. Some of the patients reported having used the herbal medicines and not found any relief of symptoms and thus preferred the modern medicines.

Regarding the practices towards glycemic control, 376 (83.2%) of the participants had good medication adherence. This finding was lower than similar studies done in Ethiopia (95% - 99%) 13,20. This was attributed to distance from the health centre for drug refills, cost of drugs, busy schedules involving travelling. Only 4.4% of the participants had a history of cigarette smoking which was lower than in Addis Ababa 12% 20 and Ethiopia 11.2% 13 15.4% of the participants had a history of drinking alcohol.

Most of the patients reported eating vegetables at least 4 times in a week and doing physical exercise at least 7 times a week. These exercises were main in the form of jogging, working to the workplace, and occupations that involved a great deal of physical activity. Of the participants, 49.6% reported to have regular blood sugar checkups. Most of the participants 192 (44.8%), had their blood sugars monitored only during the reviews in the clinics, 22.7% at least once a week, 19.8% at least once a month, 3.6% only when not feeling well and 1.6% hardly had their blood sugars monitored. This finding was attributed the fact most of the participants 60.4% did not own a glucometer and of those that owned one, some 16.9% didn't know how to use it. Some also preferred not to regularly check their blood sugar to avoid the anxiety that comes with regular checks if the blood sugars are found to be high.72.1% of the participants reported to have the body weight well controlled.

Almost all the participants, 331 (73.2%) reported to having experienced peripheral neuropathy, only 40 participants (8.8%) had had a diabetic foot ulcer and only 1 participant (0.2%) reported a cardiovascular complication. Less than half of the participants, 28.1% and 36.1% reported good eye care and foot care respectively. This study resulting being much lower than studies done in Ethiopia (43.7%)13, Iran (33%) 21 and United Arab Emirates (81.8%).^22^

From this study, good knowledge was significantly associated with current age, marital status, level of education, employment status, religion, age at diagnosis, duration with DM diagnosis, and having received training on GC. However, using multivariate logistic regression, only education level, training on glycemic control and distance from health facility were significantly associated with good knowledge. Participants with diploma, degree, and training in glycemic control and those living 17 more than 10 kilometers away from health facility were significantly more likely to have good knowledge than their respective counterparts. Our finding was consistent with studies done elsewhere reporting that high education level among participants was positively associated with good knowledge in Lebanon^23^ and Sri Lanka^16^ Bangladesh 24 This was arguably because highly educated individuals are more curious to find out more about their illness as compared to the less educated ones.^14^

Similar to a study done in the UAE 22, participants advanced in age we less likely to have good knowledge on DM indicating the need for special attention and increased care are required among patients who in most cases are also illiterate.

The management of DM largely depends on the patient's ability to do self-care in their daily lives, and therefore, patient education is always considered an essential element of DM management. Studies have shown that patients, who are knowledgeable about the DM self-care, have better long-term glycemic control 22. Knowledge about glycemic control can help the people to understand the factors associated with diabetes and motivate them to seek proper treatment and care and to keep the disease under control 25. Better glycemic management of DM requires not only the prescription of an appropriate nutritional and pharmacological regime by the physician but also intensive education of the patient 13.

### Strengths and limitations of the study

Interpretation of the results of this study can be made of the background of the following strengths and limitations. First, this study was the first to assess the KAPs toward GC of patients with DM in Uganda. The tool used to measure KAPs was adopted from previous studies^13^ ^14^ ^15^ with considerable improvements and modifications. The tool was pretested among patients from another ward to assess for clarity and comprehension of the different parameters. The data were then, collected using an interviewer-administered questionnaire to avoid misunderstanding of the questions associated with self-administered. Additionally, the interviewers in this study were fourth and final year medical students who were familiar with conducting interviews and taking patient history. This study was limited by the fact that it was carried out at only tertiary health-care facilities and was thus not necessarily be representative of the KAPS towards glycemic control elsewhere in the country or in private facilities. The KAPs question response of participants might be affected by both interviewers and recall bias.

## Conclusions

In conclusion, this study provides an insight to the current state of the KAPs towards GC of patients with DM in Uganda. More than half of the patients had good knowledge about GC. The study showed that good knowledge about GC was significantly associated with having receive training on GC, level of education and distance from the health facility.

## Future studies

This being the pioneer study of the KAPs towards GC in Uganda, future studies are still required to assess if such interventions could be effective in improving patients' KAPs towards GC as well as improving the health outcomes and quality of life of the patients living with DM in Uganda.

## Data Availability

The underlying data supporting this article and the validated questionnaire used in this study can be made available by emailing the corresponding author with reasonable request.

## Abbreviations

DM: Diabetes Mellitus
KAP: knowledge, attitude and Practice
GC: Glycemic control
MNSH: Mulago National Specialised Hospital
KNRH: Kiruddu National Referral Hospital
